# Quantitative In Situ Monitoring of Cu-Atom Release by Cu_2_O Nanocatalysts under Photocatalytic CO_2_ Reduction Conditions: New Insights into the Photocorrosion Mechanism

**DOI:** 10.3390/nano13111773

**Published:** 2023-05-31

**Authors:** Areti Zindrou, Yiannis Deligiannakis

**Affiliations:** Laboratory of Physical Chemistry of Materials & Environment, Department of Physics, University of Ioannina, 45110 Ioannina, Greece; a.zindrou@uoi.gr

**Keywords:** Cu_2_O, CuO, photocorrosion, FSP, EPR, ASV, Cu^2+^ release, CO_2_ photocatalysis, HCO_3_, surface precipitation

## Abstract

Cu_2_O is among the most promising photocatalysts for CO_2_ reduction, however its photocorrosion remains a standalone challenge. Herein, we present an in situ study of the release of Cu ions from Cu_2_O nanocatalysts under photocatalytic conditions in the presence of HCO_3_ as a catalytic substrate in H_2_O. The Cu-oxide nanomaterials were produced by Flame Spray Pyrolysis (FSP) technology. Using Electron Paramagnetic Resonance (EPR) spectroscopy in tandem with analytical Anodic Stripping Voltammetry (ASV), we monitored in situ the Cu^2+^ atom release from the Cu_2_O nanoparticles in comparison with CuO nanoparticles under photocatalytic conditions. Our quantitative, kinetic data show that light has detrimental effect on the photocorrosion of Cu_2_O and ensuing Cu^2+^ ion release in the H_2_O solution, up to 15.7% of its mass. EPR reveals that HCO_3_ acts as a ligand of the Cu^2+^ ions, promoting the liberation of {HCO_3_-Cu} complexes in solution from Cu_2_O, up to 27% of its mass. HCO_3_ alone exerted a marginal effect. XRD data show that under prolonged irradiation, part of Cu^2+^ ions can reprecipitate on the Cu_2_O surface, creating a passivating CuO layer that stabilizes the Cu_2_O from further photocorrosion. Including isopropanol as a hole scavenger has a drastic effect on the photocorrosion of Cu_2_O nanoparticles and suppresses the release of Cu^2+^ ions to the solution. Methodwise, the present data exemplify that EPR and ASV can be useful tools to help quantitatively understand the solid–solution interface photocorrosion phenomena for Cu_2_O.

## 1. Introduction

The rapid development of human society has led to an increase in energy demands and ensuing environmental deterioration, making the use of new and renewable energy sources imperative. Photocatalysts have become a research hotspot over the last decades. The pioneer work of Fujishima and Honda in 1972 [1] paved the way for light-induced water dissociation by TiO_2_ and has ignited numerous studies on photocatalysts, especially TiO_2_ [2,3]. Since then, research interest has increased exponentially, combined with the discovery of numerous photocatalysts ranging from metal oxides (e.g., ZnO [4], WO_3_ [5], and SnO_2_ [6]), non-oxides (e.g., CdS [7], CuInS_2_ [8] and ZnS [9]) and metal-free semiconductors (C_3_N_4_ [10]). Among them, Cu_2_O stands out as particularly interesting [11,12,13] thanks to its highly reducing conduction band edge located at E_CB_ = −1000 mV vs. NHE (pH = 0) [12].

Cu_2_O is a promising photocatalyst for CO_2_ [14,15,16] reduction and H_2_ production [17,18], i.e., it has a direct band-gap structure with a small energy gap of 2.0–2.2 eV [12], allowing it to absorb efficiently in the visible range of the solar spectrum, maximizing sunlight harvesting. Despite these advantages, photostability issues are among the well-documented drawbacks of Cu_2_O [13,19,20]. The so-called photocorrosion phenomenon encodes the key problem, i.e., the photogenerated holes (h^+^) and electrons (e^−^) can be adversely consumed to the self-decomposition of Cu_2_O itself [20]. At low degrees of photocorrosion, some Cu^1+^ atoms of Cu_2_O can be either oxidized to Cu^2+^ by the holes (self-photooxidation), or can be reduced to Cu^0^ atoms by the electrons (self-photoreduction) [20]. Both self-photooxidation and self-photoreduction are due to the energy positioning of the {Cu^1+^/Cu^2+^} {Cu^1+^/Cu^0^} redox couples within the band gap of Cu_2_O [21] i.e., E_1/2_{Cu^1+^/Cu^2+^} = 600 mV vs. NHE (pH = 0), E_1/2_{Cu^1+^/Cu^0^} = 470 mV vs. NHE (pH = 0) [12,13]. This phenomenon, even when it does not modify the Cu_2_O crystal structure much, limits the electron transfer from the Cu_2_O photocatalyst crystal to the surrounding acceptors or donors, which is detrimental to the photocatalytic activity [12,13,19]. It is well anticipated that when photocorrosion accumulates, physical detachment of Cu^2+^ ions can occur, resulting in severe destabilization of the Cu_2_O crystal as a whole. Toe et al. revealed that self-photooxidation is the dominant photocorrosion mechanism for Cu_2_O [19]. Practically, without the use of a hole scavenger and upon illumination, transformation of Cu_2_O to CuO occurs, with no evidence of Cu^0^ formation regardless of the presence of an electron scavenger. Moreover, in [19], XRD and SEM images confirm the growth of CuO on the surface of Cu_2_O.

In this context, the study of the photocorrosion effects of Cu_2_O under light plus CO_2_ is particularly appealing, i.e., since there is a thrust in the use of Cu_2_O as a CO_2_-reduction photocatalyst. To this end, most of the previous studies have mainly used photoelectrochemical tools to study the photocorrosion of Cu_2_O [22,23]. Complementary information on the fate of the Cu_2_O structure can be monitored with XRD [19], XPS [23] and Raman spectroscopy [24] to name a few methods. Herein, we introduce a methodology for in situ monitoring of the release of Cu^2+^ ions from Cu_2_O under photocatalytic CO_2_-reduction conditions. The method is based on in tandem use of a high analytical sensitivity method, Anodic Stripping Voltammetry (ASV) [25] and Electron Paramagnetic Resonance (EPR) spectroscopy [26]. EPR spectroscopy has been proven a valuable tool for the study of Cu^2+^ ions at the oxide–solution interface. Examples include monitoring of Cu^2+^ species in Spinel-Type Oxide Mg_1_–xCuxAl_2_O [27], Fe-doped copper oxide nanoparticles [28], Cu^2+^ on Al_2_O_3_ [29] and mononuclear Cu complexes immobilized on SiO_2_ [30]. We have demonstrated that EPR can provide detailed information on Cu^2+^ surface coordination, i.e., such as distances between neighboring Cu sites [30,31]. Thus, EPR can provide quantitative coordination information on the Cu^2+^ interaction with surfaces. Herein, we used EPR as a state-of-the-art tool to monitor in situ the formation of Cu^2+^ ions by Cu_2_O nanoparticles under photocorrosion scenarios/conditions. In addition, we used electroanalytical Anodic Stripping Voltammetry for precise analytical determination of Cu^2+^ ions released in solution [32]. Recently, we demonstrated that ASV can be used as a very sensitive analytical tool to detect trace levels (part-per-billion, ppb) of cadmium (Cd^2+^) ions released during the photocorrosion of CdS quantum dots [33]. Thus, herein, our methodology was based on the combined use of EPR and ASV to monitor the formation of Cu^2+^ ions at the Cu_2_O and their release in the reaction solution phase.

The Cu_2_O nanocatalysts used herein were synthesized using Flame Spray Pyrolysis (FSP) technology [34,35]. Previously, synthesis of CuO has been achieved by FSP by Waser et al. [36]. However, thus far, synthesis of high-purity Cu_2_O by FSP has not been achieved. Zhu et al. reported the successful existence of a Cu_2_O fraction in their CuO particles made by FSP synthesis [37]. Athanassiou et al. used a modified FSP reactor operating under highly reducing conditions to produce carbon-coated metallic copper nanoparticles [38]. Herein, in addition to Cu_2_O, we also synthesized CuO nanoparticles using Flame Spray Pyrolysis (FSP) as reference materials to study the Cu^2+^-release dynamics under the photocatalytic CO_2_-reduction process.

The specific aims of the present research were: [i] to monitor quantitatively the kinetics of Cu^2+^ ions release in solution, using EPR and ASV under photocorrosion conditions of Cu_2_O vs. CuO photocatalysts. [ii] To clarify the role of HCO_3_^−^ as substrate. [iii] To understand the role of photoinduced holes in the observed photocorrosion process.

## 2. Materials and Methods

### 2.1. Flame Spray Pyrolysis (FSP) Synthesis of CuO and Cu_2_O Nanoparticles

A conventional FSP process was used for the synthesis of CuO, as described in detail in previous works [39,40,41]. A precursor solution of 0.25 M was prepared by dissolving Copper (II) Nitrate trihydrate (Cu(NO_3_)_2_• 3H_2_O 99–104%, Sigma-Aldrich (Saint Louis, MO, USA)) in a 1:1 (by volume) mixture of acetonitrile (≥99.9%, Supelco (Bellefonte, Pennsylvania, USA)) and ethylene glycol (≥99%, Supelco (Bellefonte, PA, USA)). This precursor solution (P) was fed at a rate of P = 5 mL min^−1^ to our system and atomized to fine droplets using an oxygen dispersion flow of D = 5 L min^−1^ at a pressure drop of 1.5 bar. The spray was ignited and sustained by an oxygen/methane pilot flame of O_2_/CH_4_: 4/2 L min^−1^. For the particle collection, an additional 10 L min^−1^ O_2_ sheath was used, and the produced particles were deposited on a glass microfiber filter (Hahnemühle GF 6 257) with the assistance of a vacuum pump (BUSCH V40).

**Figure 1:**
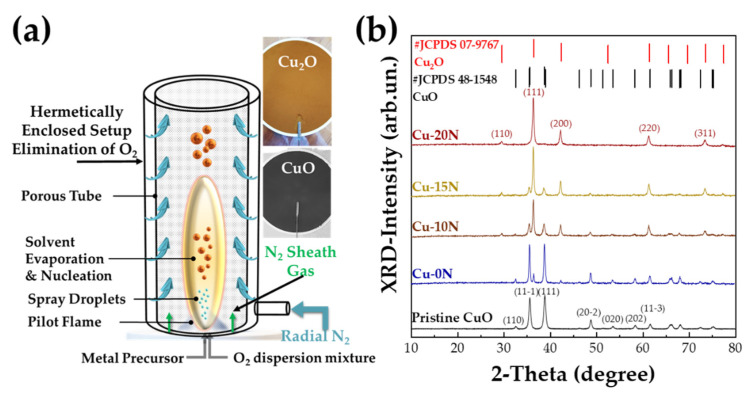
(**a**) Anoxic FSP reactor set-up used for the synthesis of CuO, Cu_2_O nanomaterials. The photos are the as-produced CuO and Cu_2_O powders on the FSP filter; (**b**) XRD patterns of our Cu-oxide nanomaterials.

The synthesis of high-purity Cu_2_O nanoparticles required a more-demanding FSP- setup with control of the combustion-atmosphere surrounding the spray nozzle (see Figure 1a). We used a cylindrical metal chamber consisting of two concentric tubes, a sinter metal tube (outer tube) and a porous metal tube (inner tube) to isolate the flame compartment from the surrounding atmosphere The porous walls of the inner tube allow the radial inflow of an inert mixing gas, in our case, N_2_, to exclude O_2_. Moreover, to provide an additional O_2_-excluding source and aid the particle collection, a 10 L min^−1^ N_2_ sheath was used. Once again, a 0.25 M precursor solution of Cu(NO_3_)_2_• 3H_2_O dissolved in a 1:1 mixture of acetonitrile and ethylene glycol was sprayed into our system with a P/D ratio of 3/3. A series of N_2_ radial inflows were tested in the range 0 to 30 L min^−1^, resulting in progressively higher Cu_2_O-phase percentages. In all cases, in addition to the radial N_2_, a N_2_ sheath gas was fixed at 10 L min^−1^, except in the case of pristine CuO, where we used a 10 L min^−1^ O_2_ sheath. The produced materials, listed in Table 1, are codenamed as Cu-xN, where x = the radial N_2_-inflow in L/min^−1^. In Table 1, we list the most pertinent materials with the Cu-20N to contain the higher Cu_2_O fraction (>95%). Higher radial N_2_ inflows resulted in the deterioration of particle crystallinity and are not discussed herein.

### 2.2. Characterization of Materials

Powder X-Ray Diffraction (pXRD): The as-prepared nanomaterials were characterized using a powder X-ray diffractometer (Bruker D8 Advanced using CuKα radiation = 1.5405 Å) with a scanning step of 0.03° at a rate of 2 s per step and 2-theta (θ) angle ranging from 10–80° at current 40 mA and voltage 40 kV. The average crystal size was calculated by using the Scherrer Equation (1) [42]:

(1) $$d_{XRD}=\frac{k\lambda }{\beta (cos\theta )}$$

where, *d_XRD_* is the crystallite size (nm), *k* is a shape constant (in this case 0.9), *λ* is the wavelength of CuKα radiation, *β* is the full width at half maximum and θ is the peak-diffraction angle. To determine the percentage of CuO/Cu_2_O crystal phase in each Cu-based nanomaterial, we used Profex, which is a graphical user interface for Rietveld refinement [43].

### 2.3. Electron Paramagnetic Resonance Spectroscopy (EPR)

EPR spectra were recorded at 77 K using a Bruker ER200D spectrometer equipped with an Agilent 5310 A frequency counter operating at X-band (~9.6 GHz) with a modulation amplitude of 10 G peak to peak. The spectrometer is controlled with a custom-made software based on LabView. To obtain an adequate signal-to-noise ratio, each spectrum is an average of 5–10 scans. Theoretical analysis of the Cu^2+^ EPR signals was performed using a spin Hamiltonian and can be simulated using EasySpin MATLAB toolbox [44] assuming a spin system with S = 1/2 and I = 3/2 for ^63,65^Cu^2+^.

### 2.4. Analytical Cu^2+^ Leaching Study by Anodic Stripping Voltammetry (ASV)

The concentration of Cu^2+^ in aqueous solution was determined by Anodic Stripping Voltammetry (ASV) using a Metrohm 797 VA computrace stand equipped with a Metrohm multimode electrode (MME). More specifically, a conventional three-electrode arrangement was used comprising Hanging Mercury Drop Electrode (HMDE) as the working electrode, Platinum rod (Pt) as the auxiliary electrode and Ag/AgCl (3 mol L^−1^ KCl) as the reference electrode. Cu standard solutions used for the quantification of our unknown samples were prepared by dissolving Cu(NO_3_)_2_• 3H_2_O in ultrapure triple-distilled (3d) water obtained from a Millipore-Q water purification system (USA) with a resistivity of >18 MΩ cm and diluting to obtain the desired Cu concentrations. The measurements were carried out at a volume of 10 mL of 0. 1 M KNO_3_ and 3 d water of pH:4 adjusted with HNO_3_ to ensure the maximum presence of Cu^2+^ ions based on the theoretical copper speciation for hydroxo complexes in pure water [45]. The instrumental settings were as follows: mercury drop size 0.4 mm and scan rate 20 mV s^−1^. Moreover, a deposition potential of −0.6 V versus Ag/AgCl (+0.2 V versus SHE at 25 °C) was used and the deposition time was carried out for 90 s. The reported data represent an average of three independent experimental repetitions.

## 3. Results

Figure 1a shows the FSP reactor set-up and photos of as-produced pure CuO and Cu_2_O powders on the FSP filter. The black color is typical for CuO, while the red-brown color of Cu_2_O originates from its band gap Eg = 2.0–2.2 eV [12]. Figure 1b shows the XRD patterns of Cu materials, also listed in Table 1. The characteristic peaks at 35.6°, 38.8° and 48.8° correspond to the planes (11-1), (111) and (20-2) of CuO (JCPDS card no. 48-1548) while the peaks at 29.6°, 36.4° and 42.3° are characteristic of the planes (110), (111) and (200) of Cu_2_O (JCPDS card no. 07-9767).

The XRD data in Figure 1 show that increasing N_2_ inflow, promoted the formation of Cu_2_O at the expense of the originally predominating CuO phase. The XRD-estimated particle diameters values (d_XRD_) of the CuO and Cu_2_O phases as well as their respective phase percentages are listed in Table 1. We see that Cu-20N is a Cu_2_O material with at least 95% and a minor fraction of CuO. Based on several trials, we conclude that a small percentage (2–5%) of CuO was formed upon exposure of the originally pure Cu_2_O to atmospheric O_2_ during the particle handling. Once formed, this CuO did not further increase. Thus, the Cu_2_O/CuO phase compositions listed in Table 1 represent stable compositions of FSP-made nanomaterials.

To underscore the Cu_2_O-formation process, we note that in FSP, the gas atmosphere where the particle formation takes place, is of key importance [34,46]. Under an oxygen-rich atmosphere, i.e., such as ambient air inflow with 20% O_2_, the produced materials are highly stable and fully oxidized ceramic powders [47]. In the present case of Cu oxide formation, this FSP protocol results in the formation of pristine CuO, see Figure 1. Decreasing the oxygen concentrations in the FSP reactor by the N_2_ sheath and mostly by the radial N_2_ inflow, see Figure 1a, resulted in the promotion of stable, reduced metal oxide (Cu_2_O) whose lattice is formed by Cu^1+^ ions. In our case, the use of N_2_ in our FSP reactor played a dual role: first, the exclusion of oxygen and second, the reduction of oxygen partial pressure inside the reactor, resulting in the progressive formation of Cu_2_O. We should note here that the formation of metallic Cu^0^ was not observed, which led us to conclude that this modified FSP setup allows meticulous exploration of the formation of suboxides rather than metallic particles.

### 3.1. Cu^2+^ Ion Release under CO_2_-Photoreduction Conditions

**Figure 2:**
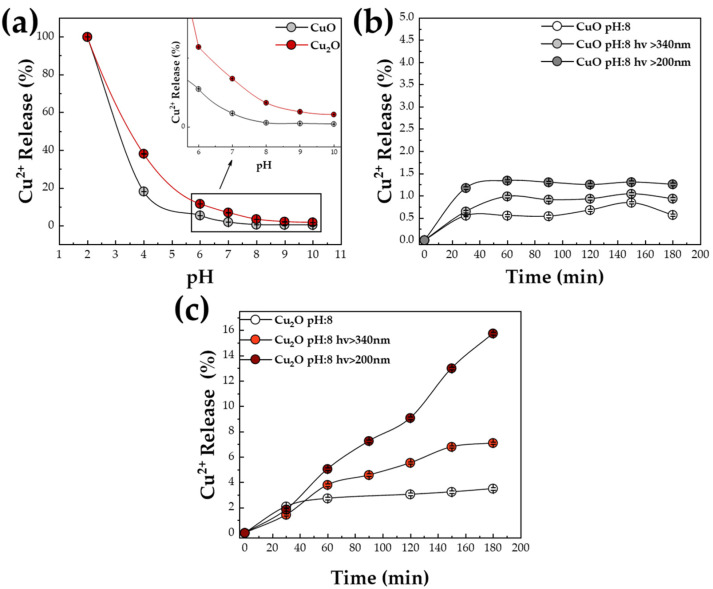
Release of Cu^2+^ atoms in solution, determined by ASV. The percentages show the fraction of Cu ions released vs. the Cu ion content of the added Cu oxide, in each experiment. (**a**) Release of Cu^2+^ ions by CuO and Cu_2_O (material Cu-20N) dissolution versus pH values, from highly acidic (pH:2) to slightly basic (pH:10), for an incubation time of 3 h. (**b**,**c**) Release of Cu^2+^ ions by CuO and Cu_2_O (material Cu-20N) in H_2_O pH:8, under the effect of Xenon-light photons (hv > 200 nm and hv > 340 nm).

*The Role of pH*: First, we examined the chemical stability, without light, by monitoring the Cu ions’ release from CuO and Cu_2_O in H_2_O under different pH values. Figure 2a presents results based on ASV determination of Cu^2+^ ions in solution after 3 h of exposure. This time scale (3 h) is typical time span for photocatalytic experiments. As we see in Figure 2a, under acidic pH (pH:2), both CuO and Cu_2_O materials were 100% dissolved after 3 h. On the contrary, increasing the pH towards more alkaline values, Cu^2+^ release decreased rapidly, with a threshold pH > 7, where the Cu^2+^ release was <5% at 3 h. Notice that the CuO phase exhibited better chemical stability than Cu_2_O. Even at neutral pH, Cu_2_O was more unstable, having a dissolution of 7%, which is 3.5-fold higher vs. the corresponding leaching of CuO (better viewed at the zoomed Figure 2a inset).

*The Role of Light-Photons:* Based on these results, a series of Xenon-lamp illuminations were performed under a slightly alkaline pH (pH:8), often used in CO_2_ photocatalysis in HCO_3_^−^/H_2_O systems [12,14], and both CuO and Cu_2_O are relatively stable, with Cu^2+^ release of 0.6% and 3.5%, respectively (Figure 2a). As seen in Figure 2b, under full-Xenon spectrum illumination, hv > 200 nm, CuO showed ~1.5% light-induced Cu^2+^ release, that is a +1% increase vs. no light. Elimination of UV photons by filtration hv > 340 nm resulted in a lower Cu^2+^ release by CuO, i.e., by ~1% (Figure 2b). Overall, the data in Figure 2a show that the damage of light on the CuO nanoparticles was limited.

On the contrary, light photons exerted a severe effect on Cu^2+^ leaching by the Cu_2_O nanophase (material Cu-20N) (Figure 2c). Full-Xenon illumination, hv > 200 nm, resulted in dissolution higher than >15% of the Cu_2_O matrices, releasing the Cu^2+^ ions in the aqueous solution. Thus, hv > 200 nm photons enhanced the Cu release by 500%, i.e., from ~3% in the dark to ~15%. Filtering out the UV photons, hv > 340 nm, resulted in a significant drop of Cu^2+^ ions release to 7% (Figure 2c), which is about 200% versus no light. Overall, the data in Figure 2b,c reveal that [i] Cu_2_O is far more prone, about 10 fold, to Cu^2+^ release in solution than CuO. [ii] This is a direct manifestation of photocorrosion. That is to say, photocorrosion starts as an oxidation event inside the Cu_2_O crustal, as evidenced by many previous data [19,20], and, in the following, the present data show that photocorrosion persists until the physical detachment of the Cu ions from the particle matrix. As we show hereafter, photoinduced holes are the origin of the Cu^1+^ to Cu^2+^ oxidation.

The effect of photon wavelength can be understood as follows: the band gap of Cu_2_O particles near 2.1 eV entails that photons with λ ≤ 580 nm, i.e., visible and UV photons, can photoexcite it, creating holes and electrons. This includes 200 nm photons, i.e., hv~6 eV, which excite highly energetic “deep” holes with energies well below the valence band top. Similarly, electrons well above the conduction-bend edge can be excited. The data in Figure 2c, with hv > 200 nm, indicate that the high energetic holes dramatically boost the Cu^2+^ release. This results in a significant 15% of the Cu_2_O mass to literally deteriorate. In the same context, allowing hv > 340 nm contains photons with energy ≤ 3.4 eV that can also photoexcite “deep” holes, though with less energy than the 200 nm photons. Thus, the hv > 340 nm results in about half of the Cu^2+^ release by the Cu_2_O particles.

**Figure 3:**
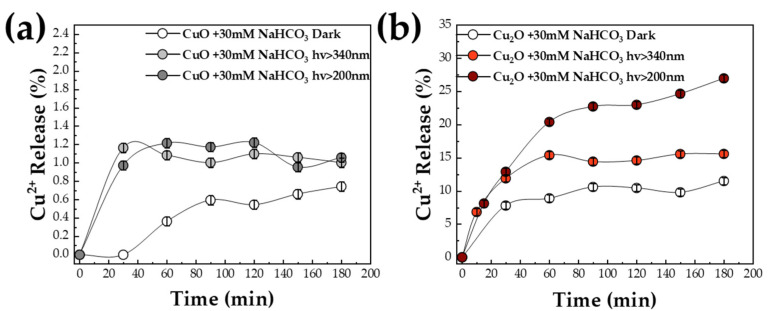
Release of Cu^2+^ ions in solution, in the presence of 30 mM NaHCO_3_, determined by ASV. (**a**) % of Cu^2+^-ions release by CuO versus time, in H_2_O pH 8, in the dark or under illumination. (**b**) % of Cu^2+^-ions release by Cu_2_O (material Cu-20N) in H_2_O pH:8 versus time, in dark or under illumination (hv > 200 nm and hv > 340 nm).

*The Role of HCO_3_^−^:* As mentioned previously [48,49], Cu_2_O is identified as a promising CO_2_ photocatalyst. In aqueous-phase photocatalytic processes, carbonate species are pertinent. Herein, we tested the role of HCO_3_*^−^* as a photocatalytic substrate that prevails in the pH range 6.5–10.5 in H_2_O systems [50]. We used 30 mM HCO_3_^−^, which is an average amount used in CO_2_-photocatalytic experiments [51,52]. Control data show that HCO_3_*^−^* with no illumination had an insignificant effect on Cu^2+^ release (Figure 3a) from CuO. Similarly, the Cu^2+^ release data in Figure 3a show that during underexposure of CuO in HCO_3_*^−^* plus light, Cu-atom release was extremely low, i.e., 0.75% without irradiation and ~1% with hv > 200 nm. This confirms the stability of CuO under light and as well as light +HCO_3_*^−^*.

In the case of Cu_2_O, the presence of HCO_3_^−^ alone with no light (Figure 3b) caused a Cu-atom release ~11%. This is higher than the Cu^2+^ release by Cu_2_O with no HCO_3_*^−^*, i.e., 3.5% (compare Figure 3b vs. Figure 2c). This reveals a direct chemical, not photochemical effect of HCO_3_*^−^* on the Cu_2_O atoms. As we show hereafter by EPR data, HCO_3_*^−^* extracts Cu^2+^ ions from the Cu_2_O particles s via formation of Cu-HCO_3_ complexes.

As seen in Figure 3b, under light photons, the HCO_3_^−^ severely intensifies the Cu^2+^ release, which reached ~27% of its mass (Figure 3b) under hv > 200 nm. Filtering off UV photons (Figure 3b), hv > 340 nm, resulted in ~15% Cu^2+^ release. These results clearly reveal that carbonate, i.e., HCO_3_^−^ exerts a deteriorating effect in two ways: [i] In the dark, HCO_3_^−^ is able to drive detachment of some Cu atoms from the Cu_2_O particles. [ii] Under illumination, the photocorrosive Cu release is exacerbated by the presence of carbonates.

### 3.2. EPR Spectroscopy

**Figure 4:**
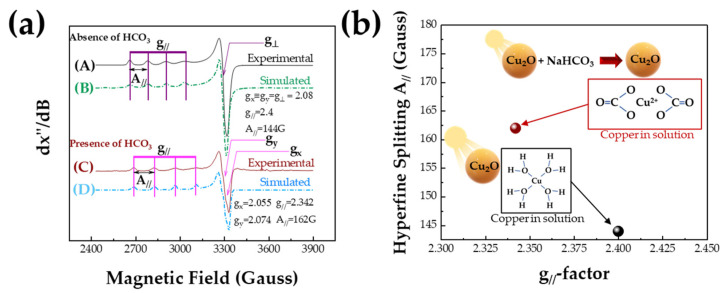
(**a**) 77K-EPR spectra for Cu^2+^ ions release from irradiated Cu_2_O particles in the absence of the presence of 30 mM NaHCO_3_. The same samples as those used in Figure 2 and Figure 3 and were used. (Solid lines: experimental EPR spectra, dotted lines: theoretical simulations of Cu^2+^-EPR using the Spin Hamiltonian parameter listed in Table 2). (**b**) The relation between g_//_ and A_//_ parameters for Cu^2+^ ions in the presence and absence of HCO_3_^−^.

Figure 4a shows X-band EPR spectra for Cu^2+^ ions released by Cu_2_O particles under Xenon light irradiation, either in the presence or absence of HCO_3_. All spectra displayed in Figure 4a are typical for mononuclear Cu^2+^ (electron spin *S* = 1/2, nuclear spin *I* = 3/2) [30,53]. The well-resolved hyperfine lines of Cu^2+^ EPR spectra correspond to isolated Cu^2+^ ions in solution. All EPR spectra can be simulated, assuming a spin system with *S* = 1/2, *I* = 3/2, i.e., for Cu^2+^, see dotted lines in Figure 4a with Cu^2+^ Spin Hamiltonian parameters (tensors g and A), listed in Table 2. In Figure 4b, we represent a so-called Peisach–Blumberg plot [54] for Cu^2+^ species using the g_//_ and A_//_ from Table 2. Peisach and Blumberg developed a method which correlates EPR parameters (g_//_, A_//_) with the number and type of ligand donor atoms in Cu^2+^ complexes. Previously, we showed that this method may be used to precisely detect the coordination of Cu^2+^ ions on metal oxides’ surfaces and to distinguish the form of Cu atoms in solution [30,31].

The structural significance of the EPR spectral features can be understood by comparison of the g_//_ and A_//_ parameters with the literature data according to the method established by Peisach and Blumberg. These data indicate that: (a) In the absence of carbonates, the Cu^2+^ ions are released from illuminated Cu_2_O as aqua-coordinated ions in solution. (b) In the presence of HCO_3_^−^ as a photocatalytic substrate, copper ions are released in the form of Cu(HCO_3_^−^)_2_ complexes in the aqueous solution. In all cases, the Cu^2+^ ions are coordinated by O atoms in an octahedral symmetry with the ground-state orbital of the Cu-unpaired electron to be $d_{x^{2}-y^{2}}$ [55,56].

## 4. Discussion

The present data show that in the presence of HCO_3_^−^, the Cu_2_O photocorrosion is severely accentuated. Even in the dark, bicarbonate should be viewed as a highly active coordinating agent that can bind on the Cu_2_O surface and promote the release of Cu (HCO_3_^−^)_2_ complexes in aqueous solution. Additionally, light photons can promote the formation of Cu^2+^ via self-oxidation.

**Figure 5:**
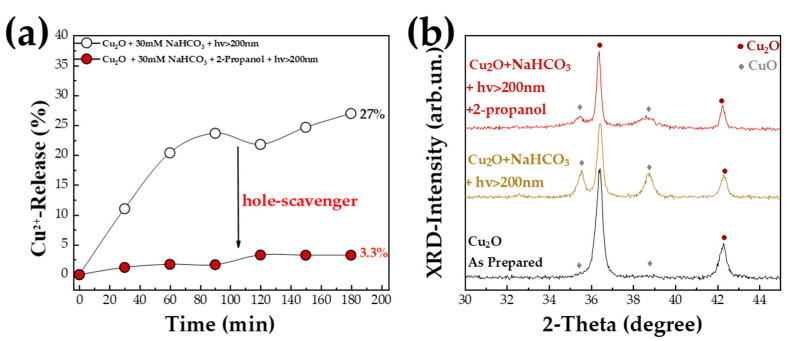
(**a**) % of Cu^2+^-ions release by Cu_2_O (material Cu-20N) versus irradiation time (hv > 200 nm) in H_2_O pH 8, in the presence of NaHCO_3_ or NaHCO_3_ plus 2-propanol. (**b**) XRD patterns by Cu_2_O (material Cu-20N) after 3 h irradiation in presence of NaHCO_3_ or NaHCO_3_ plus 2-propanol.

*The Role of Hole Scavenger:* The data in Figure 2 and Figure 3 clearly exemplify the photocorrosion phenomena that prevail in Cu_2_O. As mentioned by Toe [19,20], photoinduced holes should be considered as the key reactive species that promote the Cu^2+^ release from photo-cited Cu_2_O. In Figure 5, we examine the role of hole scavenger using isopropanol as a standard hole scavenger.

In the presence of 2-propanol plus NaHCO_3_, a significant suppression of the photocorrosion is observed, as evidenced by the decrease from 27% to 3% of Cu^2+^-ion release (Figure 5a). This provides clear evidence that scavenging of the photoinduced holes, provides significant protection against photocorrosion of Cu_2_O under realistic CO_2_-photocatalytic conditions. This is a very encouraging result, showing a route to address the Cu_2_O photocorrosion problem.

To further understand the process, we examined by XRD the Cu_2_O particles after 3 h photocatalytic exposure (Figure 5b). As seen in Figure 5b, in the presence of NaHCO_3_, after 3 h of irradiation (Xenon, hv > 200 nm) the initial Cu_2_O-crystal phase composition is changed from >95% Cu_2_O (see Table 3) to 60% CuO. We underline that the particles collected after 3 h photocorrosion represent only the fraction that is not dissolved to Cu^2+^ ions. Thus, the photocorrosion of Cu_2_O in the presence of NaHCO_3_ has two consequences: [i] Part of the Cu_2_O particle is dissolved towards Cu^2+^ ions. [ii] The remaining Cu-oxide particle phase is altered from Cu_2_O to CuO. Importantly, in the presence of 2-propanol, the Cu^2+^-release and XRD data show that [i] Practically minimal Cu^2+^-ions release occurs. That is the Cu-oxide particles remain mostly intact. [ii] The crystal composition is modified, i.e., according to Table 3, the Cu-oxide particles consist of 25% CuO, i.e., the initial 95% Cu_2_O has been retained to 75%. We consider that the formed 25% CuO forms a protective layer around the Cu_2_O, and this inhibits the Cu^2+^-ion release.

## 5. Conclusions

Using EPR spectroscopy in tandem with ASV, the in situ study of the release of Cu ions from Cu_2_O nanocatalyst under photocatalytic conditions provides new insight into the role of HCO_3_ as a catalytic substrate. Light and HCO_3_^−^ have detrimental effects on the photocorrosion of Cu_2_O and the ensuing Cu^2+^-ion release in the H_2_O solution. EPR reveals that HCO_3_^−^ acts as ligand of the Cu^2+^ ions, promoting the liberation of {HCO_3_-Cu} complexes in solution from Cu_2_O, up to 27% of its mass. Even in the dark, bicarbonate acts as a highly active coordinating agent that can bind on Cu_2_O surface and promote the release of Cu (HCO_3_^−^)_2_ complexes in aqueous solution. On top of this, light photons can promote the formation of Cu^2+^ via self-oxidation. XRD data show that under prolonged irradiation, part of Cu^2+^ ions can re-precipitate on the Cu_2_O surface, creating a passivating CuO layer that stabilizes the CuO-Cu_2_O from further photocorrosion. Moreover, including isopropanol as a hole scavenger has a drastic impact on the photo-oxidation of Cu_2_O to CuO as well as suppresses the release of Cu^2+^ ions. Method-wise, the present data exemplify that EPR and ASV can be useful tools to quantitatively understand the solid–solution interface photocorrosion phenomena for Cu_2_O.

## Figures and Tables

**Table 1:** Structural characteristics of the FSP-made Cu-oxide nanomaterials.

	Radial N_2_ (L min^−1^)	CuO (%)	Cu_2_O (%)	d_XRD_ CuO (nm)	d_XRD_ Cu_2_O (nm)
Pristine CuO	-	100 ± 1	-	20 ± 1	-
Cu-0N	0	90 ± 2	10 ± 2	29 ± 1	34 ± 1
Cu-10N	10	60 ± 2	40 ± 2	21 ± 1	30 ± 1
Cu-15N	15	40 ± 3	60 ± 2	22 ± 1	31 ± 1
Cu-20N (Cu_2_O)	20	5 ± 3	95 ± 2	-	25 ± 1

**Table 2:** Spin Hamiltonian EPR parameters used to simulate the Cu^2+^-EPR spectra for the atoms released from Cu_2_O nanomaterials.

	g [g_x_, g_y_, g_z_]	A_z_ = A_//_Gauss	Reference
Cu^2+^ from Cu_2_O + hv (Xenon > 200 nm)	g_x_ = g_y_ = g_˔⊥_ = 2.08 g_z_ = g_//_ = 2.4	144	This work
Cu^2+^ from Cu_2_O + hv (Xenon > 200 nm) + NaHCO_3_	g_x_ = 2.055 g_y_ = 2.074 g_z_ = g_//_ = 2.342	162	This work
Cu^2+^ + H_2_O (pH:2)	g_x_ = 2.078 g_y_ = 2.078 g_z_ = g_//_ = 2.42	126	[29]
(Cu^2+^ in zeolites) Cu-CHA hydrated	g_x_ = g_y_ = g_˔⊥_ = 2.07 g_z_ = g_//_ = 2.394	157	[57]
(Cu^2+^ in zeolites) Cu-MOR hydrated	g_x_ = g_y_ = g_˔⊥_ = 2.08 g_z_ = g_//_ = 2.4	154	[57]

**Table 3:** XRD analysis of Cu_2_O particles before and after the photocatalytic/photocorrosion process.

Material	CuO (%)	Cu_2_O (%)	d_XRD_ CuO (nm)	d_XRD_ Cu_2_O (nm)
Cu_2_O (Cu-20N)	5 ± 3	95 ± 3	-	25 ± 1
Cu_2_O + 30 mM NaHCO_3_ + hv > 200 nm	60 ± 3	40 ± 3	17 ± 1	26 ± 1
Cu_2_O + 30 mM NaHCO_3_ + hv > 200 nm + 2-propanol	25 ± 3	75 ± 3	8 ± 1	33 ± 1

## Data Availability

Not applicable.
